# The application of collaborative nursing model improve the postoperative pulmonary rehabilitation in patients with lung carcinoma: a retrospective study of 345 cases

**DOI:** 10.3389/fmed.2026.1843501

**Published:** 2026-06-17

**Authors:** Qian Li, Dechao Zhang

**Affiliations:** 1 Lung Cancer Center/Lung Cancer Institute, West China Hospital, Sichuan University/West China School of Nursing, Sichuan University, Chengdu, Sichuan, China; 2 Department of Critical Care Medicine, West China Hospital, Sichuan University/West China School of Nursing, Sichuan University, Chengdu, Sichuan, China

**Keywords:** lung neoplasms, nursing, psychological wellbeing, quality of life, rehabilitation

## Abstract

**Introduction:**

Lung carcinoma is a leading cause of cancer-related mortality worldwide, and while surgical resection remains a primary treatment option, postoperative recovery often presents significant challenges, particularly in terms of pulmonary function and quality of life (QOL). The study aimed to evaluate the effectiveness of the Collaborative Nursing Model (CNM) in improving postoperative pulmonary rehabilitation.

**Methods:**

This retrospective study included 345 lung carcinoma patients who underwent surgical resection. Patients were divided into an intervention group (n = 248), which received postoperative care under the CNM, and a control group (n = 97), which received standard postoperative care. Outcomes assessed included pulmonary function recovery, rehabilitation adherence, QOL, postoperative complications, and psychological wellbeing.

**Results:**

The intervention group showed greater improvements in pulmonary function compared with the control group, including FVC (1 +6.0%, 95% CI 3.6–8.4), FEV1 (1 +5.9%, 95% CI 3.4–8.4), and MVV (1 +4.93 L/min, 95% CI 3.4–6.5). Rehabilitation adherence (84.8% vs. 62.3%; RD +22.5%, 95% CI 12.1–32.9%) and dietary adherence (88.3% vs. 70.4%; RD +17.9%, 95% CI 7.8–28.0%) were higher in the intervention group. Greater improvements were also observed in quality of life, with larger reductions in SGRQ scores (1 –7.44, 95% CI –10.2 to –4.7) and increases in SF-36 scores (1 +7.94, 95% CI 5.7–10.2). The incidence of pulmonary infections was lower in the intervention group (8.2% vs. 17.3%; RD –9.1%, 95% CI –17.8 to –0.4), while differences in other complications were not statistically significant. Psychological outcomes were improved, with greater reductions in anxiety and depression scores (both 1 –1.97, 95% CI –3.0 to –1.0).

**Discussion:**

The CNM was associated with improved postoperative pulmonary rehabilitation outcomes in lung cancer patients, including better pulmonary function, higher adherence, improved QOL, and a lower incidence of complications and psychological distress.

## Introduction

As one of the most significant health challenges worldwide, lung carcinoma is characterized by high incidence, morbidity, and mortality rates, accounting for approximately 11.4% of all cancer diagnoses and 18.0% of cancer-related deaths globally, making it the leading cause of cancer-related mortality (1, 2). In China alone, more than 800,000 new cases are diagnosed annually, placing a substantial burden on healthcare systems (3). According to the Global Burden of Disease (GBD) study, lung cancer contributes substantially to the global disease burden, accounting for over 40 million disability-adjusted life years (DALYs), reflecting its profound impact on both premature mortality and long-term morbidity (1, 4). Despite advances in early detection and multimodal treatment strategies, lung cancer survival rates remain low, particularly for those diagnosed at advanced stages (5, 6). Surgery, particularly lobectomy or pneumonectomy, remains the cornerstone treatment for early-stage lung cancer, offering the best chance for long-term survival. In addition, emerging technologies are rapidly advancing early cancer detection and prognosis. Nanoscale diagnostic tools enable highly sensitive detection of tumor biomarkers, while machine learning-based models can integrate complex clinical and imaging data to improve early prediction and risk stratification. These approaches provide new opportunities for more precise and personalized management of lung cancer (7, 8). However, the invasive nature of these procedures poses considerable challenges for postoperative recovery, particularly concerning pulmonary function.

Surgical resection of lung tissue, although effective in removing malignant tumors, leads to substantial alterations in pulmonary structure and function (9). Postoperative complications, such as decreased lung capacity, impaired gas exchange, and weakened respiratory muscles, are common (9, 10). Studies have shown that lung function parameters, such as forced vital capacity (FVC), forced expiratory volume in 1 s (FEV1), and maximal voluntary ventilation (MVV), can decrease by as much as 30–50% in the immediate postoperative period (11, 12). These impairments can persist for months and significantly limit patients’ physical activity, leading to reduced exercise tolerance and quality of life (QOL) (13). Furthermore, complications like pulmonary infections, atelectasis, and prolonged dyspnea further hinder recovery, especially in patients with pre-existing comorbidities or advanced age. To mitigate these issues, pulmonary rehabilitation has become an integral component of postoperative care. Traditionally, pulmonary rehabilitation programs focus on improving lung function through structured exercise regimens, respiratory muscle training, and nutritional support (14, 15). Nevertheless, adherence to these programs remains suboptimal due to various barriers, including limited patient motivation, inadequate education, and lack of coordinated care among healthcare providers (16). This highlights the need for innovative approaches to improve the effectiveness and accessibility of pulmonary rehabilitation in lung cancer patients.

Nursing care plays a pivotal role in supporting lung cancer patients undergoing surgical treatment (17). Nurses are often the primary point of contact for patients throughout their surgical journey, providing essential care during the preoperative, intraoperative, and postoperative phases. Preoperatively, nurses focus on educating patients about the surgical procedure, managing anxiety, and optimizing baseline pulmonary function. Postoperatively, their role extends to monitoring for complications, managing symptoms, and encouraging early mobilization and pulmonary exercises. Effective nursing care is crucial for reducing the risk of complications, promoting recovery, and improving patients’ overall outcomes (18). Currently, traditional nursing models often operate in silos, limiting communication and collaboration among multidisciplinary teams. This fragmented approach can result in inconsistent care delivery, reduced patient satisfaction, and suboptimal outcomes. In recent years, the concept of collaborative care has gained attention as a means to address these shortcomings and enhance the quality of nursing care.

The Collaborative Nursing Model (CNM) represents an interdisciplinary approach to healthcare delivery, emphasizing teamwork and communication among nurses, physicians, patients, and their families (19, 20). Rooted in principles of patient-centered care, CNM aims to align the efforts of all stakeholders to achieve common goals. Key components of the model include comprehensive patient education, tailored care plans, shared decision-making, and regular follow-up (21, 22). By fostering a sense of shared responsibility, CNM has been reported to be associated with enhanced patient satisfaction and a reduced incidence of adverse events. In the context of pulmonary rehabilitation for lung cancer patients, CNM provides a structured framework for integrating various interventions into a cohesive care plan. For instance, nurses can collaborate with physiotherapists to design individualized exercise programs, provide psychological support to address anxiety and depression, and involve family members in the rehabilitation process to ensure continuity of care at home (23). Moreover, CNM leverages evidence-based practices to standardize care delivery, thereby minimizing variability and enhancing outcomes. Despite its potential benefits, the application of CNM in postoperative pulmonary rehabilitation remains underexplored, particularly in the field of lung cancer treatment. While existing studies have highlighted the effectiveness of interdisciplinary approaches in managing chronic respiratory diseases such as chronic obstructive pulmonary disease (COPD) (24, 25), limited data are available on their impact in the surgical oncology setting. This knowledge gap underscores the need for further research to evaluate the feasibility and efficacy of CNM in improving pulmonary rehabilitation outcomes for lung cancer patients.

Given the significant burden of postoperative complications and the limitations of conventional rehabilitation programs, there is an urgent need to explore innovative strategies for optimizing care delivery in lung cancer patients. In the current study, we retrospectively evaluated the application of CNM in the postoperative pulmonary rehabilitation of 345 lung carcinoma patients. By analyzing outcomes such as pulmonary function recovery, adherence to rehabilitation protocols, and QOL improvements, this study aims to evaluate the association between CNM and postoperative pulmonary rehabilitation outcomes in this population. The findings will not only contribute to the growing body of literature on collaborative care models but also inform clinical practice and policy-making, paving the way for broader implementation of CNM in surgical oncology.

## Materials and methods

### Participants and experiment design

This study retrospectively analyzed pulmonary function in 345 lung cancer patients who underwent surgical treatment and received nursing care between 2019 and December 2021. The inclusion criteria were as following: Adults aged 18 years and older diagnosed with lung carcinoma and undergoing surgical treatment; Patients who completed a minimum of 6 months of postoperative follow-up; Availability of complete medical records, including pulmonary function tests and quality-of-life assessments. Tumor staging in this study was based on postoperative pathological staging. A small proportion of stage IV patients were included, reflecting cases with oligometastatic disease or those undergoing palliative or cytoreductive surgical interventions. The exclusion criteria were as follows: Patients with severe cognitive impairments or psychiatric conditions. Those who declined participation in postoperative rehabilitation programs. Individuals with incomplete medical records. Baseline demographic and clinical characteristics were further extracted from medical records, including age, sex, smoking history, smoking burden (pack-years), comorbidities, presence of chronic obstructive pulmonary disease (COPD), tumor stage, surgical approach [video-assisted thoracoscopic surgery (VATS) or thoracotomy], type of resection (lobectomy or pneumonectomy), and preoperative pulmonary function parameters [forced vital capacity (FVC), forced expiratory volume in one second (FEV1), and maximal voluntary ventilation (MVV)].

Patients were classified into two groups based on the nursing strategies received. Intervention group: patients receiving postoperative care under the CNM, which included structured respiratory function training, education and counseling, supervised exercise programs, dietary interventions, and family involvement. Control Group: patients receiving standard postoperative care, which consisted of routine nursing practices, such as basic health education, monitoring for complications, and general physical activity advice without tailored or multidisciplinary interventions. This study was approved by the institutional review board of West China Hospital, which covered the entire study period including extended data collection. Due to the retrospective design, the requirement for written informed consent was waived by the ethics committee.

### Nursing strategy

**FIGURE 1:**
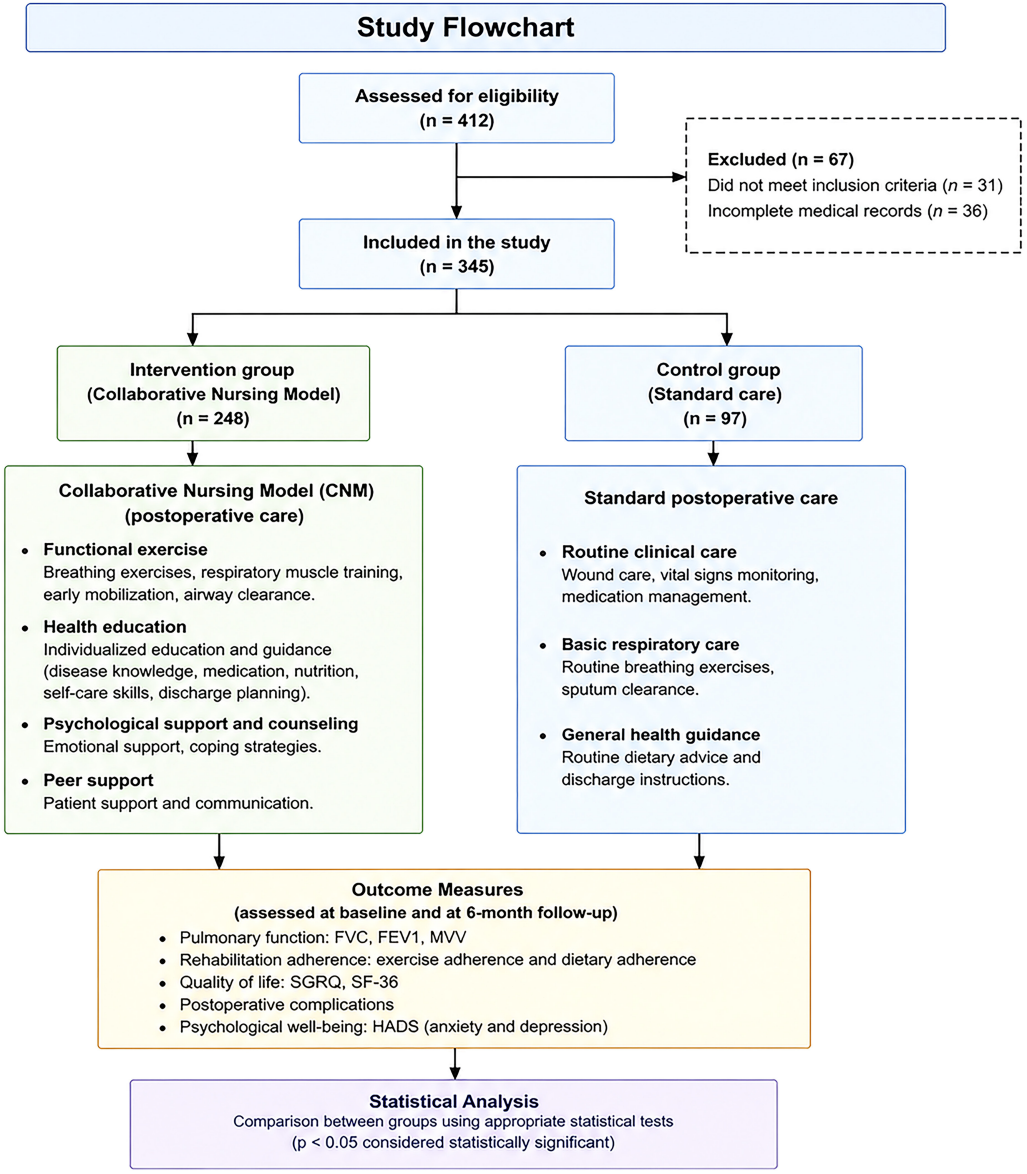
Study flowchart and framework of the collaborative nursing model (CNM). This figure illustrates the retrospective study design and the structure of the CNM intervention. Patients were screened, included, and classified into intervention and control groups based on nursing strategy. The CNM integrates multidisciplinary collaboration, respiratory training, exercise programs, dietary support, psychological care, and family involvement. Outcome measures include pulmonary function (FVC, FEV1, MVV), rehabilitation adherence, quality of life (SGRQ, SF-36), postoperative complications, and psychological wellbeing (HADS). FVC, forced vital capacity (FVC); FEV1, forced expiratory volume in 1 s (FEV1); MVV, maximal voluntary ventilation (MVV). SGRQ, Saint George’s Respiratory Questionnaire; SF-36, Short Form Health Survey. HADS, hospital Anxiety and Depression Scale (HADS).

The CNM applied in this study followed a structured framework, as illustrated in Figure 1. The model was nurse-led and involved multidisciplinary collaboration among physicians, nurses, patients, and family members. The intervention integrated respiratory function training, health education, exercise programs, dietary support, psychological care, and family participation, aiming to improve pulmonary function, rehabilitation adherence, quality of life, and psychological wellbeing. The CNM intervention was designed as a comprehensive and interdisciplinary approach to postoperative pulmonary rehabilitation, focusing on enhancing physical recovery, emotional support, and overall quality of life. The intervention consisted of the following key components: (1) Respiratory function training. Patients were provided with structured breathing exercises designed to enhance lung capacity and strengthen respiratory muscles. These exercises included diaphragmatic breathing, pursed-lip breathing, and inspiratory muscle training. Progress was monitored using spirometry, and adjustments to the training were made based on individual progress and tolerance. Sessions were conducted daily under the supervision of trained respiratory therapists and nurses. (2) Education and counseling. Educational sessions were conducted to empower patients with knowledge about their condition, the importance of adherence to rehabilitation protocols, and strategies for symptom management. Topics included recognizing early signs of complications, proper use of respiratory equipment, and lifestyle modifications. Psychological counseling sessions were provided to address anxiety, depression, and overall emotional wellbeing, with peer support groups providing a sense of community and shared experience. (3) Exercise programs. Tailored physical activity regimens were implemented, including supervised walking programs, resistance training, and flexibility exercises. The goal was to improve overall physical fitness, enhance endurance, and reduce the risk of deconditioning. Patients were encouraged to increase their activity levels gradually, with periodic assessments to track progress. (4) Dietary interventions. Nutritionists developed personalized meal plans emphasizing high-protein, anti-inflammatory foods to promote tissue repair and reduce inflammation. Nutritional counseling included guidance on managing weight, maintaining adequate hydration, and incorporating essential vitamins and minerals into daily meals. Supplementation was provided when necessary to address specific deficiencies. (5) Family involvement. Recognizing the importance of family support, the intervention included family-centered education and training sessions. Family members were taught how to assist patients with breathing exercises, encourage adherence to rehabilitation protocols, and provide emotional support. This collaborative approach ensured that patients received consistent care both in clinical settings and at home. A detailed comparison of the components and implementation of routine care and the collaborative nursing model is provided in Table 1 to ensure reproducibility.

**TABLE 1:** Comparison of routine care and collaborative nursing model (CNM).

Component	Routine care	Collaborative nursing model (CNM)
Care structure	Standard nursing	Multidisciplinary (nurse-led team with physician and family involvement)
Respiratory training	Basic instruction only	Structured program (daily breathing exercises: diaphragmatic, pursed-lip, inspiratory muscle training)
Exercise program	General advice	Supervised, individualized exercise plan (progressively adjusted)
Education	Basic discharge education	Structured education sessions + continuous counseling
Psychological support	Not routinely provided	Regular psychological counseling + emotional support
Dietary intervention	General dietary advice	Individualized nutrition plan + dietitian involvement
Family involvement	Minimal	Active participation + training for home support
Adherence monitoring	Not standardized	Regular monitoring (attendance, compliance tracking)
Follow-up intensity	Routine follow-up	Structured follow-up with feedback and adjustment

### Outcome measures

The effectiveness of the CNM intervention was evaluated using outcome domains consistent with the framework illustrated in Figure 1, including pulmonary function recovery, rehabilitation adherence, quality of life, postoperative complications, and psychological wellbeing.

#### Primary outcomes

Pulmonary function recovery: Lung function was assessed using key metrics such as forced vital capacity (FVC), forced expiratory volume in 1 s (FEV1), and maximal voluntary ventilation (MVV). Measurements were taken at baseline (pre-intervention) and during follow-up, with the primary analysis focusing on the 6-month postoperative outcomes. Although assessments at 1 and 3 months were conducted in routine practice, these data were not included in the present analysis. Rehabilitation Adherence: Adherence was measured through attendance records at rehabilitation sessions, completion rates of prescribed exercises, and self-reported adherence to dietary recommendations and home-based activities. Quality of Life (QOL): Patient-reported QOL was evaluated using validated scales, such as the Saint George’s Respiratory Questionnaire (SGRQ) and the Short Form Health Survey (SF-36). These scales captured dimensions including physical functioning, emotional wellbeing, and social participation.

#### Secondary outcomes

Incidence of postoperative complications: The occurrence of complications such as pulmonary infections, atelectasis, and prolonged dyspnea was tracked during the follow-up period. The severity and resolution of these complications were documented. Psychological wellbeing: Levels of anxiety and depression were assessed using standardized tools, such as the Hospital Anxiety and Depression Scale (HADS). These assessments provided insights into the emotional impact of the rehabilitation process and the effectiveness of psychological support components. The integration of these outcome measures ensured a comprehensive evaluation of the CNM intervention, capturing both physiological and psychosocial dimensions of recovery.

### Statistical analysis

Data were analyzed using SPSS software. Descriptive statistics, such as means and standard deviations, were employed to summarize baseline characteristics and outcomes for both the intervention and control groups. To evaluate differences between pre- and post-intervention outcomes within each group, paired t-tests were conducted. Continuous variables were expressed as mean ± standard deviation and compared using independent-samples *t*-tests, while categorical variables were expressed as number (percentage) and compared using χ^2^ tests or Fisher’s exact tests. Baseline comparability between the two groups was assessed using these methods. Effect sizes and 95% confidence intervals were calculated where applicable. Multivariate analysis of variance (MANOVA) was performed to assess the effect of multiple dependent variables (e.g., pulmonary function, QOL) simultaneously while accounting for potential confounding factors such as age, gender, and baseline health status. To enhance the robustness of the analyses, propensity score matching (PSM) was performed to reduce baseline imbalance between the intervention and control groups. The propensity score was estimated using a multivariable logistic regression model. A 1:1 nearest-neighbor matching algorithm without replacement was applied. Covariate balance was assessed using standardized mean differences (SMD), with values < 0.1 indicating acceptable balance. A significance level of *p* < 0.05 was set for all analyses, with 95% confidence intervals reported where applicable.

## Results

### Patient characteristics

A total of 345 patients with lung carcinoma who underwent surgical resection between January 2019 and December 2021 were included in the study. Patients were retrospectively grouped according to the period of care in routine clinical practice. Specifically, patients treated before implementation of the CNM were included in the control group, whereas those treated after CNM implementation were included in the intervention group. The two groups were comparable in terms of baseline demographic and clinical characteristics (Table 2). As shown in Table 2, sex distribution was explicitly reported, with males accounting for 60.5 and 61.9% of patients in the intervention and control groups, respectively. No significant difference in sex distribution was observed between groups (*p* = 0.911), indicating that potential sex-related bias was minimized. In addition, most baseline demographic and clinical characteristics were comparable between the two groups (all *p* > 0.05). However, a statistically significant difference was observed in surgical approach, with a higher proportion of patients undergoing VATS in the control group compared to the intervention group (*p* = 0.010). This variable was therefore considered in subsequent analyses to reduce potential confounding. This reduces the likelihood that observed differences were driven by baseline imbalance. The unequal sample sizes between the two groups (248 vs. 97) reflect real-world clinical practice, including differences in patient volume across time periods and the phased implementation of the CNM. The overall study workflow and intervention structure are summarized in Figure 1.

**TABLE 2:** Baseline characteristics of the study population.

Variable	Intervention (*n* = 248)	Control (*n* = 97)	Effect size (95% CI)	Test statistic	*p*-value	SMD
Age (years)	63.4 ± 8.2	64.1 ± 7.8	−0.7 (−2.5 to 1.1)	*t* = −0.87	0.388	−0.10
Sex (male/female)	150 (60.5%)/98 (39.5%)	60 (61.9%)/37 (38.1%)	–	χ^2^ = 0.01	0.911	−0.03
Smoking history [n (%)]	153 (62%)	58 (60%)	2.0% (−9.5 to 13.5%)	χ^2^ = 1.10	0.293	0.04
Smoking burden (pack-years)	35.74 ± 10.10	34.64 ± 10.11	1.10 (−1.3 to 3.5)	*t* = 0.91	0.364	0.11
Comorbidities [n (%)]	80 (32%)	28 (31%)	1.0% (−10.2 to 12.2%)	χ^2^ = 0.00	0.978	0.02
COPD (%)	61 (24.6%)	31 (32.0%)	−7.4% (−18.5 to 3.7%)	χ^2^ = 1.57	0.212	−0.17
Tumor stage (III/IV)	124 (50.0%)/124 (50.0%)	52 (53.6%)/45 (46.4%)	−3.6% (−15.0 to 7.8%)	χ^2^ = 0.23	0.629	−0.07
Surgical approach (VATS/Thoracotomy)	137 (55.2%)/111 (44.8%)	69 (71.1%)/28 (28.9%)	−15.9% (−27.0 to −4.8%)	χ^2^ = 6.67	0.013	−0.33
Resection type (Lobectomy/Pneumonectomy)	179 (72.2%)/69 (27.8%)	68 (70.1%)/29 (29.9%)	2.1% (−8.8 to 13.0%)	χ^2^ = 0.06	0.802	0.05
Preoperative FVC	63.4 ± 8.5	64.2 ± 9.1	−0.8 (−2.7 to 1.1)	*t* = 1.30	0.196	−0.09
Preoperative FEV1	58.42 ± 9.26	59.86 ± 9.60	−1.44 (−3.7 to 0.8)	*t* = −1.26	0.212	−0.15
Preoperative MVV (L/min)	82.48 ± 4.94	82.79 ± 5.94	−0.31 (−1.6 to 1.0)	*t* = −0.46	0.648	−0.06

Continuous variables are presented as mean ± standard deviation and compared using independent-samples *t*-tests. Categorical variables are presented as counts (percentages) and compared using χ^2^ tests. Effect sizes are reported as mean differences (MD) or risk differences (RD) with 95% confidence intervals. Standardized mean differences (SMD) were used to assess covariate balance after propensity score matching.

### Pulmonary function measurements

The primary outcome of the study was the recovery of pulmonary function, as measured by FVC, FEV1, and MVV. At 6 months postoperatively, the intervention group demonstrated greater improvements compared with the control group. Specifically, FVC increased from 63.4 to 75.3% in the intervention group and from 64.2 to 70.1% in the control group, with a between-group difference in change (Δ) of +6.0% (95% CI 3.6–8.4, *p* < 0.001). FEV1 increased from 58.6 to 71.1% in the intervention group and from 59.2 to 65.8% in the control group, corresponding to a between-group difference of +5.9% (95% CI 3.4–8.4, *p* < 0.001). MVV increased from 82.12 to 91.23 L/min in the intervention group and from 83.43 to 87.61 L/min in the control group, with a between-group difference of +4.93 L/min (95% CI 3.4–6.5, *p* < 0.001) (Table 3). These findings suggest that the CNM was associated with greater improvements in pulmonary function.

**TABLE 3:** Effects of pulmonary function recovery of lung carcinoma patients.

Variable	Intervention Pre-Op	Intervention 6 months	Control Pre-Op	Control 6 months	*t*	*p-*value	Δ (Intervention vs. control) (95% CI)
FVC (%)	63.4 ± 8.5	75.3 ± 6.4^*,#^	64.2 ± 9.1	70.1 ± 7.3*	*t* = 5.2	< 0.001	+6.0 (3.6–8.4)
FEV1 (%)	58.6 ± 9.2	71.1 ± 8.7^*,#^	59.2 ± 9.5	65.8 ± 7.9*	*t* = 4.8	< 0.001	+5.9 (3.4–8.4)
MVV (L/min)	82.12 ± 5.22	91.23 ± 4.54^*,#^	83.43 ± 5.73	87.61 ± 4.12*	*t* = 6.1	< 0.001	+4.93 (3.4–6.5)

Data are presented as mean ± standard deviation. Within-group comparisons were performed using paired *t*-tests, and between-group differences in change (Δ) were compared using independent-samples *t*-tests. Effect sizes are reported as mean differences with 95% confidence intervals.

“*” *p* < 0.05 vs. Pre-Op;

“#” *p* < 0.05 vs. Control.

### Rehabilitation adherence measurement

Adherence to rehabilitation protocols was higher in the intervention group, indicating better engagement with the structured care provided under the CNM. Specifically, 84.8% of patients in the intervention group attended at least 80% of rehabilitation sessions compared with 62.3% in the control group, corresponding to a risk difference (RD) of +22.5% (95% CI 12.1–32.9%, *p* < 0.001). In addition, 88.3% of patients in the intervention group adhered to dietary recommendations compared with 70.4% in the control group, with an RD of +17.9% (95% CI 7.8–28.0%, *p* = 0.001) (Table 4).

**TABLE 4:** Effects of CNM strategy on rehabilitation adherence of lung carcinoma patients.

Outcome	Intervention (%)	Control (%)	χ^2^	*p*-value	RD (95% CI)
Adherence to rehabilitation	84.80%	62.30%	χ^2^ = 12.6	< 0.001	+22.5% (12.1–32.9%)
Adherence to dietary recommendations	88.30%	70.40%	χ^2^ = 10.2	0.001	+17.9% (7.8–28.0%)

Categorical variables are presented as percentages and compared using χ^2^ tests. Effect sizes are reported as risk differences (RD) with 95% confidence intervals.

### Quality of life (QOL)

QOL was assessed using the SGRQ and the SF-36 Health Survey. At 6 months, the intervention group demonstrated greater improvements compared with the control group. The SGRQ score decreased from 52.41 to 37.17 in the intervention group and from 53.12 to 45.32 in the control group, with a between-group difference in change of −7.44 (95% CI −10.2 to −4.7, *p* < 0.001). The SF-36 score increased from 52.28 to 68.51 in the intervention group and from 51.88 to 60.17 in the control group, corresponding to a between-group difference of +7.94 (95% CI 5.7–10.2, *p* < 0.001) (Table 5). These results suggest that CNM was associated with improved QOL.

**TABLE 5:** Effects of CNM strategy on quality of life of lung carcinoma patients.

Outcome	Intervention Pre-Op	Intervention 6 Months	Control Pre-Op	Control 6 months	*t*	*p*-value	Δ (95% CI)
SGRQ total score	52.41 ± 7.58	37.17 ± 5.76^*,#^	53.12 ± 8.03	45.32 ± 6.33*, #	*t* = −6.4	< 0.001	−7.44 (−10.2 to −4.7)
SF-36 total score	52.28 ± 6.41	68.51 ± 5.93*, #	51.88 ± 6.16	60.17 ± 5.13*, #	*t* = 7.1	< 0.001	+7.94 (5.7–10.2)

Data are presented as mean ± standard deviation. Within-group comparisons were performed using paired *t*-tests, and between-group differences in change (Δ) were compared using independent-samples *t*-tests. Effect sizes are reported as mean differences with 95% confidence intervals.

“*” *p* < 0.05 vs. Pre-Op;

“#” *p* < 0.05 vs. Control.

### Postoperative complications

Postoperative complications were also evaluated under different nursing strategies. The intervention group had a lower incidence of pulmonary infections compared with the control group (8.2% vs. 17.3%), corresponding to a risk difference of −9.1% (95% CI −17.8 to −0.4, *p* = 0.038). Although lower rates of atelectasis (5.8% vs. 12.3%) and dyspnea (4.7% vs. 9.8%) were observed in the intervention group, these differences did not reach statistical significance (*p* = 0.084 and *p* = 0.12, respectively) (Table 6). Overall, the findings suggest that the CNM was associated with a lower incidence of certain postoperative complications.

**TABLE 6:** Effects of CNM strategy on postoperative complications of lung carcinoma patients.

Outcome	Intervention (%)	Control (%)	χ^2^	*p*-value	RD (95% CI)
Pulmonary infections	8.20%	17.30%	χ^2^ = 4.3	0.038	−9.1% (−17.8 to −0.4%)
Atelectasis	5.80%	12.30%	χ^2^ = 3.0	0.084	−6.5% (−13.9 to 0.9%)
Dyspnea	4.70%	9.80%	χ^2^ = 2.4	0.12	−5.1% (−11.6 to 1.4%)

Categorical variables are presented as percentages and compared using χ^2^ tests. Effect sizes are reported as risk differences (RD) with 95% confidence intervals.

### Psychological wellbeing

Psychological outcomes were also assessed. The intervention group showed greater reductions in both anxiety and depression scores compared with the control group. Anxiety scores decreased from 10.21 to 5.56 in the intervention group and from 10.09 to 7.41 in the control group, corresponding to a between-group difference in change of −1.97 (95% CI −3.0 to −1.0, *p* < 0.001). Similarly, depression scores decreased from 9.28 to 5.21 in the intervention group and from 9.13 to 7.03 in the control group, with a between-group difference of −1.97 (95% CI −3.0 to −1.0, *p* < 0.001) (Table 7). These results suggest that CNM was associated with improved psychological wellbeing.

**TABLE 7:** Effects of CNM strategy on psychological wellbeing of lung carcinoma patients.

Outcome	Intervention Pre-Op	Intervention 6 months	Control Pre-Op	Control 6 months	*t*	*p*-value	Δ (95% CI)
Anxiety (HADS)	10.21 ± 3.52	5.56 ± 2.72^*,#^	10.09 ± 3.61	7.41 ± 3.12^*,#^	*t* = −4.1	< 0.001	−1.97 (−3.0 to −1.0)
Depression (HADS)	9.28 ± 3.17	5.21 ± 2.52^*,#^	9.13 ± 3.32	7.03 ± 3.02^*,#^	*t* = −4.2	< 0.001	−1.97 (−3.0 to −1.0)

Data are presented as mean ± standard deviation. Within-group comparisons were performed using paired *t*-tests, and between-group differences in change (Δ) were compared using independent-samples *t*-tests. Effect sizes are reported as mean differences with 95% confidence intervals. “*” *p* < 0.05 vs. Pre-Op; “#” *p* < 0.05 vs. Control.

### Subgroup analysis

Subgroup analysis was performed based on postoperative pathological staging and age. The results showed consistent improvements in pulmonary function across subgroups. In stage III patients, the intervention group demonstrated a greater increase in FVC compared with the control group (16.2% vs. 8.7%), corresponding to a mean difference of +7.5% (95% CI 4.8–10.2, *p* < 0.001). In stage IV patients, the corresponding values were 14.5% versus 7.1%, with a mean difference of +7.4% (95% CI 4.6–10.2, *p* < 0.001). Among patients aged > 70 years, FVC improvement was also greater in the intervention group (15.4% vs. 9.3%), with a mean difference of +6.1% (95% CI 2.8–9.4, *p* = 0.001) (Table 8). These findings suggest that the associations between CNM and pulmonary function improvement were consistent across clinically relevant subgroups.

**TABLE 8:** Subgroup analysis of the effects of CNM strategy.

Subgroup	Outcome	Intervention	Control	*t*	*p*-value	MD (95% CI)
Stage III	FVC change (%)	16.2 ± 5.8*	8.7 ± 6.2	*t* = 5.1	< 0.001	+7.5 (4.8–10.2)
Stage IV	FVC change (%)	14.5 ± 6.2*	7.1 ± 6.3	*t* = 4.9	< 0.001	+7.4 (4.6–10.2)
Age > 70	FVC change (%)	15.4 ± 6.5*	9.3 ± 7.3	*t* = 3.4	0.001	+6.1 (2.8–9.4)

Continuous variables are presented as mean ± standard deviation and compared using independent-samples *t*-tests. Effect sizes are reported as mean differences (MD) with 95% confidence intervals.

“*” *p* < 0.05 vs. Control.

### PSM sensitivity analysis

To further reduce potential confounding and strengthen the robustness of the findings, PSM was performed using baseline demographic, clinical, tumor-related, surgical, and pulmonary function variables. A total of 97 matched pairs were obtained using 1:1 nearest-neighbor matching without replacement. After matching, baseline imbalance, particularly in surgical approach, was substantially improved, with most standardized mean differences reduced to below 0.1 (Supplementary Table S1). In the matched cohort, the intervention group remained associated with superior outcomes compared with the control group. Specifically, greater improvements were observed in pulmonary function parameters, including FVC (Δ +8.37%, 95% CI 5.8–10.9), FEV1 (Δ +5.18%, 95% CI 2.9–7.5), and MVV (Δ +5.20 L/min, 95% CI 3.7–6.7) (Supplementary Table S2). Rehabilitation adherence (RD +22.7%, 95% CI 11.5–33.9%) and dietary compliance (RD +18.2%, 95% CI 8.4–28.0%) were also higher (Supplementary Table S3). Improvements in quality of life were observed, with reductions in SGRQ (Δ−7.48, 95% CI −10.2 to −4.8) and increases in SF-36 (Δ +7.84, 95% CI 5.6–10.1) (Supplementary Table S4). Psychological outcomes were similarly improved, with greater reductions in anxiety and depression scores (Supplementary Table S6), while lower rates of complications were observed (Supplementary Table S5). Subgroup analyses further confirmed consistency across tumor stage and age groups (Supplementary Table S7). These findings indicate that the observed associations were robust after adjustment for baseline differences.

## Discussion

The present study evaluated the effectiveness of the CNM framework in improving postoperative pulmonary rehabilitation outcomes in lung cancer patients. However, given the retrospective design of this study, these findings should be interpreted as associations rather than causal effects. The results demonstrated that this structured, multidisciplinary intervention was associated with significant improvements in pulmonary function, rehabilitation adherence, quality of life, and psychological wellbeing, and a lower incidence of complications. The analysis results showed that patients in the CNM intervention group experienced significantly greater improvements in pulmonary function (FVC, FEV1, and MVV), rehabilitation adherence, and QOL compared to the control group, which received standard postoperative care. Additionally, the intervention group had fewer postoperative complications, including pulmonary infections, atelectasis, and prolonged dyspnea, and reported greater reductions in anxiety and depression levels. These findings suggest that the CNM may be associated with improved recovery outcomes for lung cancer patients following surgery. By focusing on both the physical and psychosocial aspects of recovery, CNM may represent a comprehensive approach that addresses common barriers to postoperative recovery and rehabilitation.

The results of our study are consistent with a growing body of literature that emphasizes the importance of integrated, multidisciplinary care models in improving postoperative outcomes for lung cancer patients. Previous studies have highlighted the benefits of pulmonary rehabilitation programs (PRP) in enhancing pulmonary function, physical activity, and QOL in patients recovering from lung cancer surgery (26). A meta-analysis by Lu et al. (27) demonstrated that structured PRP significantly improves pulmonary function in patients following lung cancer surgery, particularly in terms of FEV1 and FVC (27). Likewise, studies have shown that enhancing QOL through rehabilitation interventions is crucial for long-term recovery, as poor postoperative QOL can negatively affect overall survival rates and patient satisfaction (28, 29). However, few studies have explored the impact of the CNM strategy specifically in postoperative pulmonary rehabilitation, making this study one of the first to address this gap.

Our findings are in line with the study by Tenconi et al. (30), which explored the effects of a similar interdisciplinary approach in lung cancer patients. Their study found significant improvements in pulmonary function and QOL when a team of physicians, nurses, physiotherapists, and dietitians collaborated in postoperative care (30). In contrast, our study provides a more detailed look at the specific role of nursing, education, and psychological support within CNM, highlighting the integrated efforts of the nursing team in guiding the rehabilitation process. This distinction sets our study apart, emphasizing how nursing professionals play a critical role in improving adherence to rehabilitation protocols and managing patient emotions throughout the recovery journey. The psychological benefits of the CNM observed in our study also align with findings from previous research on the psychological challenges faced by lung cancer patients after surgery. A study by Powell et al. (31) demonstrated that anxiety and depression are prevalent in patients post-lung cancer surgery, which can significantly hinder recovery (31). Our results showing a marked reduction in both anxiety and depression scores in the intervention group (*p* < 0.001) underline the importance of psychological support as part of a comprehensive rehabilitation program. Unlike many previous studies that focus on physical rehabilitation alone, our study integrated psychological support through counseling and peer group sessions, which we believe contributed to the observed improvements in emotional wellbeing.

To address potential confounding inherent in the retrospective design, propensity score matching was performed using a comprehensive set of baseline variables. After matching, baseline imbalance—particularly in surgical approach—was substantially reduced, improving comparability between groups. In the matched cohort, the intervention group remained associated with better pulmonary function recovery, higher rehabilitation adherence, improved quality of life, and more favorable psychological outcomes, while also demonstrating a lower incidence of postoperative complications. These consistent findings across multiple clinically relevant domains suggest that the observed associations are robust and not solely attributable to baseline differences. However, several important considerations should be acknowledged. Although most covariates achieved acceptable balance after matching, slight residual imbalance persisted in smoking burden and COPD status, which may still introduce some degree of confounding. In addition, propensity score matching can only account for measured variables, and the influence of unmeasured confounders cannot be excluded. Therefore, the results should be interpreted as associations rather than causal effects. Taken together, the PSM-based sensitivity analysis reinforces the primary findings of this study and supports the potential clinical value of the collaborative nursing model, while highlighting the need for prospective, randomized studies to further validate these observations.

One of the main strengths of our study is that it supports the hypothesis that this holistic model was associated with better outcomes for lung cancer patients, as evidenced by improved pulmonary function, increased rehabilitation adherence, and better QOL. Additionally, the large sample size and the retrospective design allows evaluation of real-world clinical outcomes but also limits causal inference. This may allow for more generalizable results across a wider patient population, compared to smaller, single-center studies. This study has several limitations: due to the retrospective, non-randomized design and time-based grouping, potential selection bias and residual confounding cannot be fully excluded. First, the retrospective design introduces potential bias, particularly in patient selection. Although we attempted to minimize this bias by comparing outcomes between two well-matched groups, the absence of randomization means that residual confounders may still exist. For example, the patients in the intervention group may have been more motivated or had better access to healthcare resources, which could have influenced their outcomes independently of the intervention. A prospective, randomized controlled trial (RCT) would offer stronger evidence by controlling for these variables. Another limitation is the lack of long-term follow-up. While we observed significant improvements in pulmonary function and QOL at 6 months, the long-term sustainability of these gains remains unclear. Future studies should include extended follow-up periods (e.g., 12 months or more) to assess whether the benefits of CNM are maintained over time. Additionally, an assessment of cost-effectiveness would be beneficial, as integrating a multidisciplinary team into routine care could increase healthcare costs. Evaluating the economic feasibility of CNM is essential for determining its broader applicability in diverse clinical settings. Furthermore, although our study shows that psychological support is beneficial, we did not explore the specific mechanisms through which it works. Future studies could delve deeper into how psychological interventions, such as cognitive-behavioral therapy (CBT) or mindfulness training, contribute to the improvements in emotional wellbeing observed in this study. Understanding which elements of the psychological support provided in CNM are most effective could help refine the model further.

Given the promising findings of this study, future research should aim to refine and extend the CNM model to further enhance its effectiveness. Studies exploring the specific roles of different team members (e.g., nurses, physiotherapists, dieticians) could identify which aspects of the intervention are most influential. In addition, longitudinal studies are needed to assess the long-term impact of CNM on survival rates, recurrence, and quality of life. Future trials should also consider examining the potential cost-effectiveness of CNM, especially as healthcare systems worldwide strive to implement more efficient models of care. Moreover, it would be valuable to conduct studies that incorporate patient-reported outcomes (PROs) to understand patients’ subjective experiences of rehabilitation. Collecting qualitative data from patients could provide valuable insights into the aspects of CNM that patients find most helpful, as well as any challenges they face during recovery. Such insights could inform adjustments to the model to make it even more patient-centered.

In conclusion, this retrospective study suggests that the CNM was associated with improved pulmonary function, rehabilitation adherence, quality of life, and psychological wellbeing, along with a lower incidence of postoperative complications in lung cancer patients. However, these findings should be interpreted with caution, as causal relationships cannot be established in this retrospective study. Although limitations exist, the study offers promising results that could influence future care models for lung cancer patients and other surgical populations.

## Data Availability

The raw data supporting the conclusions of this article will be made available by the authors, without undue reservation.
