# Supplementary material for: The application of collaborative nursing model improve the postoperative pulmonary rehabilitation in patients with lung carcinoma: a retrospective study of 345 cases
Source: Front Med (Lausanne). 2026 Jun 17;13:1843501. doi: 10.3389/fmed.2026.1843501 (PMC13319056; doi:10.3389/fmed.2026.1843501)
Supplement: Supplementary file 1 [file Data_Sheet_1.docx]

**Table S1 Baseline characteristics after propensity score matching**

| **Variable** | **Intervention (n = 97)** | **Control (n = 97)** | **Effect size (95% CI)** | **Test statistic** | ***p* value** | **SMD** |
| --- | --- | --- | --- | --- | --- | --- |
| Age (years) | 64.71 ± 7.02 | 64.08 ± 7.23 | 0.63 (-1.3 to 2.5) | t = 0.61 | 0.542 | 0.09 |
| Sex (Male/Female) | 59 (60.8%) / 38 (39.2%) | 60 (61.9%) / 37 (38.1%) | - | χ² = 0.02 | 0.889 | -0.02 |
| Smoking history [n (%)] | 64 (66.0%) | 63 (64.9%) | 1.1% (-10.0–12.2%) | χ² = 0.02 | 0.887 | 0.02 |
| Smoking burden (pack-years) | 33.57 ± 9.34 | 34.64 ± 10.11 | -1.07 (-3.5 to 1.4) | t = -0.77 | 0.444 | -0.11 |
| Comorbidities [n (%)] | 30 (30.9%) | 29 (29.9%) | 1.0% (-10.2–12.2%) | χ² = 0.02 | 0.887 | 0.02 |
| COPD (%) | 39 (40.2%) | 31 (32.0%) | 8.2% (-3.6–20.0%) | χ² = 1.46 | 0.227 | 0.17 |
| Tumor stage (III/IV) | 49 (50.5%) / 48 (49.5%) | 52 (53.6%) / 45 (46.4%) | -3.1% (-14.5–8.3%) | χ² = 0.18 | 0.669 | -0.06 |
| Surgical approach (VATS/Thoracotomy) | 56 (57.7%) / 41 (42.3%) | 69 (71.1%) / 28 (28.9%) | -13.4% (-25.2–-1.6% | χ² = 3.51 | 0.061 | -0.28 |
| Resection type (Lobectomy/Pneumonectomy) | 69 (71.1%) / 28 (28.9%) | 68 (70.1%) / 29 (29.9%) | 1.0% (-9.9–11.9%) | χ² = 0.02 | 0.889 | 0.02 |
| Preoperative FVC (%) | 60.69 ± 7.05 | 62.02 ± 9.20 | -1.33 (-3.6 to 0.9) | t = -1.15 | 0.252 | -0.16 |
| Preoperative FEV1 (%) | 61.25 ± 8.85 | 59.86 ± 9.60 | +1.39 (-1.2 to 4.0) | t = 1.07 | 0.287 | 0.15 |
| Preoperative MVV (L/min) | 83.17 ± 5.02 | 82.79 ± 5.94 | +0.38 (-1.0 to 1.8) | t = 0.49 | 0.625 | 0.07 |

Continuous variables are presented as mean ± standard deviation and compared using independent-samples t-tests. Categorical variables are presented as counts (percentages) and compared using χ² tests. Effect sizes are reported as mean differences (MD) or risk differences (RD) with 95% confidence intervals. Standardized mean differences (SMD) were used to assess covariate balance after propensity score matching.

**Table S2 Effects of pulmonary function recovery after propensity score matching (n =97 for each group)**

| **Variable** | **Intervention Pre-Op** | **Intervention 6 Months** | **Control Pre-Op** | **Control 6 Months** | **t** | ***p* value** | **Δ (Intervention vs Control) (95% CI)** |
| --- | --- | --- | --- | --- | --- | --- | --- |
| FVC (%) | 60.69 ± 7.05 | 76.85 ± 6.12*^, #^ | 62.02 ± 9.20 | 69.81 ± 6.94* | t = 7.5 | <0.001 | +8.37 (5.8–10.9) |
| FEV1 (%) | 61.25 ± 8.85 | 72.27 ± 8.01*^, #^ | 59.86 ± 9.60 | 65.70 ± 7.55* | t = 5.3 | <0.001 | +5.18 (2.9–7.5) |
| MVV (L/min) | 83.17 ± 5.02 | 91.91 ± 4.33*^, #^ | 82.79 ± 5.94 | 86.33 ± 4.01* | t = 9.3 | <0.001 | +5.20 (3.7–6.7) |

Data are presented as mean ± standard deviation. Within-group comparisons were performed using paired t-tests, and between-group differences in change (Δ) were compared using independent-samples t-tests. Effect sizes are reported as mean differences with 95% confidence intervals. “*” *p* < 0.05 vs. Pre-Op; “#” *p* < 0.05 vs. Control.

**Table S3 Effects of CNM strategy on rehabilitation adherence after propensity score matching (n =97 for each group)**

| **Outcome** | **Intervention (%)** | **Control (%)** | **χ²** | ***p* value** | **RD (95% CI)** |
| --- | --- | --- | --- | --- | --- |
| Rehabilitation adherence | 86.60% | 63.90% | χ² = 12.9 | <0.001 | +22.7% (11.5–33.9%) |
| Dietary adherence | 89.70% | 71.50% | χ² = 10.6 | 0.001 | +18.2% (8.4–28.0%) |

Categorical variables are presented as percentages and compared using χ² tests. Effect sizes are reported as risk differences (RD) with 95% confidence intervals.

**Table S4 Effects of CNM strategy on quality of life after propensity score matching (n =97 for each group)**

| **Outcome** | **Intervention Pre-Op** | **Intervention 6 Months** | **Control Pre-Op** | **Control 6 Months** | **t** | ***p* value** | **Δ (Intervention vs Control) (95% CI)** |
| --- | --- | --- | --- | --- | --- | --- | --- |
| SGRQ | 51.92 ± 7.21 | 36.58 ± 5.42*, # | 52.73 ± 7.84 | 44.87 ± 6.01*, # | t = -7.0 | <0.001 | -7.48 (-10.2 to -4.8) |
| SF-36 | 52.01 ± 6.22 | 69.12 ± 5.64*, # | 51.67 ± 6.03 | 60.94 ± 5.22*, # | t = 7.6 | <0.001 | +7.84 (5.6–10.1) |

Data are presented as mean ± standard deviation. Within-group comparisons were performed using paired t-tests, and between-group differences in change (Δ) were compared using independent-samples t-tests. Effect sizes are reported as mean differences with 95% confidence intervals. “*” *p* < 0.05 vs. Pre-Op; “#” *p* < 0.05 vs. Control.

**Table S5 Effects of CNM strategy on postoperative complications aftre propensity score matching (n =97 for each group)**

| **Outcome** | **Intervention (%)** | **Control (%)** | **χ²** | ***p* value** | **RD (95% CI)** |
| --- | --- | --- | --- | --- | --- |
| Pulmonary infections | 9.30% | 16.50% | χ² = 2.7 | 0.1 | -7.2% (-15.8–1.4%) |
| Atelectasis | 6.20% | 11.30% | χ² = 1.7 | 0.19 | -5.1% (-12.8–2.6%) |
| Dyspnea | 5.10% | 9.30% | χ² = 1.4 | 0.23 | -4.2% (-10.8–2.4%) |

Categorical variables are presented as percentages and compared using χ² tests. Effect sizes are reported as risk differences (RD) with 95% confidence intervals.

**Table S6 Effects of CNM strategy on psychological well-being after propensity score matching (n =97 for each group)**

| **Outcome** | **Intervention Pre-Op** | **Intervention 6 Months** | **Control Pre-Op** | **Control 6 Months** | **t** | **P-value** | **Δ (95% CI)** |
| --- | --- | --- | --- | --- | --- | --- | --- |
| Anxiety (HADS) | 10.12 ± 3.31 | 5.41 ± 2.51*, # | 10.05 ± 3.47 | 7.18 ± 2.94*, # | t = -5.0 | <0.001 | -1.84 (-2.8 to -0.9) |
| Depression (HADS) | 9.11 ± 3.05 | 5.02 ± 2.33*, # | 9.02 ± 3.14 | 6.82 ± 2.81*, # | t = -5.1 | <0.001 | -1.89 (-2.8 to -1.0) |

Data are presented as mean ± standard deviation. Within-group comparisons were performed using paired t-tests, and between-group differences in change (Δ) were compared using independent-samples t-tests. Effect sizes are reported as mean differences with 95% confidence intervals. “*” *p* < 0.05 vs. Pre-Op; “#” *p* < 0.05 vs. Control.

**Table S7 Subgroup analysis after propensity score matching (n =97 for each group)**

| **Subgroup** | **Outcome** | **Intervention** | **Control** | **t** | **P-value** | **MD (95% CI)** |
| --- | --- | --- | --- | --- | --- | --- |
| Stage III | ΔFVC (%) | 15.8 ± 5.4* | 8.9 ± 6.1 | t = 5.4 | <0.001 | +6.9 (4.1–9.7) |
| Stage IV | ΔFVC (%) | 14.1 ± 6.0* | 7.3 ± 6.4 | t = 5.2 | <0.001 | +6.8 (3.9–9.7) |
| Age > 70 | ΔFVC (%) | 15.1 ± 6.1* | 9.5 ± 7.0 | t = 3.5 | 0.001 | +5.6 (2.3–8.9) |

Continuous variables are presented as mean ± standard deviation and compared using independent-samples t-tests. Effect sizes are reported as mean differences (MD) with 95% confidence intervals.“*” *p* < 0.05 vs. Control.
